# On the Temperature of Man within the Tropics

**Published:** 1851-10

**Authors:** John Davy


*On the Temperature of Man within the Tropics.* By Dr. John Davy.—In continuation of some former researches on the Temperature of Man [Phil. Trans., 1845], Dr. Davy has communicated to the Royal Society the results of his subsequent observations on this subject, during a period of three years and a half, chiefly at Barbadoes, where the mean annual temperature of the atmosphere, he states, is 80° Fahr., and the range of temperature throughout the year from about 10° to 18° in the open air. The observations were made three times a day; the temperature of the body being noted, with that of the external air, the pulse, and the number of respirations per minute; all of which are duly set forth in elaborate tables. The chief general results are the following: 1. That the average temperature of man, within the tropics, is a little higher, nearly 1°, than in a temperate climate, such as that of England. 2. That within the tropics as in cooler regions, the temperature of the body is almost constantly fluctuating. 3. That the order of fluctuating is different from that in a cooler climate; the minimum degree being early in the morning, after a night's rest,—and not at night. 4. That all exertion, whether of body or mind, except itbe very gentle, has a heightening effect on the temperature; while passive exercise, especially carriage exercise, has a lowering tendency. 5. That heavy clothing, especially if tight and close, tends to raise the temperature unduly, especially under active exercise; and that close, ill-ventilated rooms, especially when crowded, have, in a marked manner, the same tendency. 6. That when the body is in a healthy state, it rapidly recovers its normal condition as to temperature. 7. That when laboring under disease, however slight, the temperature is abnormally elevated, its undue degree being some criterion of the intensity of the diseased action. 8. That within the tropics, there is comparatively little difference of temperature between the surface of the body and the internal parts; the skin is more active in its functions, and the kidneys are less active; the former state being connected, perhaps with a rapid desquamation of cuticle; the latter, with an absence of lithic acid. 9. That the effect of wine, unless used in great moderation, is commonly lowering as to temperature, whilst it accelerates the heart's action; followed, after awhile, by an increase of temperature. 10. The tendency of sea-sickness, like that of disease, is to elevate the temperature. 11. The tendency of a sea-voyage, apart from sea-sickness, is to equalize the temperature without permanently elevating it. 12. That even at sea, with a change of atmospheric temperature, there is a tendency to change of temperature of the body, increasing towards the tropics. The most interesting facts, however, are the changes of temperature depending on changes of health or disease, and the lowering influence of wines and ordinary stimulants.—*Philosophical Transactions*, 1850.—*Ibid.*

